# Empowering agricultural research: A comprehensive custard apple (*Annona squamosa*) disease dataset for precise detection

**DOI:** 10.1016/j.dib.2024.110078

**Published:** 2024-01-19

**Authors:** Sandip Thite, Kailas Patil, Rohini Jadhav, Yogesh Suryawanshi, Prawit Chumchu

**Affiliations:** a Vishwakarma University, Pune, India; b Kasetsart University, Sriracha, Thailand; c Bharati Vidyapeeth College of Engineering, Pune, India

**Keywords:** Classification, Dataset, Disease detection, Image analysis, Leaf diseases, Machine learning, Custard apple, Sugar apple, Precision agriculture

## Abstract

The Custard Apple, known as sugar apple or sweetsop, spans diverse regions like India, Portugal, Thailand, Cuba, and the West Indies. This dataset holds 8226 images of Custard Apple (Annona squamosa) fruit and leaf diseases, categorized into six types: Athracnose, Blank Canker, Diplodia Rot, Leaf Spot on fruit, Leaf Spot on leaf, and Mealy Bug. It's a key resource for refining machine learning algorithms focused on detecting and classifying diseases in Custard Apple plants. Utilizing methods like deep learning, feature extraction, and pattern recognition, this dataset sharpens automated disease identification precision. Its extensive range suits testing and training disease identification techniques. Public access fosters collaboration, fast-tracking plant pathology advancements and supporting Custard Apple plant sustainability. This dataset fosters collaborative efforts, aiding disease prevention techniques to boost Custard Apple yield and refine farming. It enhances disease identification, monitoring, and management in Custard Apple production, aiming to elevate agricultural practices and crop yields.

Specifications Table

**Table:** 

Subject	Applied Machine Learning, Agriculture
Specific subject area	Agronomy & Crop Science
Data format	Raw
Type of data	Image
Data collection	The data collection process for the "Custard Apple Disease Dataset" was meticulously carried out in the *Nimgaon-Bhogi region, located in Taluka- Shirur, Pune district, Maharashtra, India.* To ensure the dataset's relevance and diversity, images were collected from various Custard Apple plantations in the *Nimgaon-Bhogi* region, considering different growth stages, environmental conditions, and disease manifestations. The data collection process encompassed several stages to ensure a comprehensive representation of Custard apple fruit and leaf samples. The data contain images of different diseases which includes Athracnose, Blank Canker, Diplodia Rot, Leaf Spot on fruit, Leaf Spot on leaf, and Mealy Bug. The captured images were saved in JPG format and resized to a resolution of 1024 × 768 pixels. Extensive field surveys were conducted to gather a diverse range of fruit and leaves affected by various diseases. High-resolution images of Custard apple leaves were captured using quality cameras, employing multiple angles to capture different perspectives of the leaves. This included capturing images from both sides of the leaves and fruit to capture a holistic view of their condition.
Data source location	Nimgaon Bhogi, Taluka- Shirur, Dist -Pune Pin - 412220. Maharashtra, Country- India. Latitude- 18.817435, Longitude- 74.256013
Data accessibility	Repository name: **Sugar Apples / Custard Apples (Annona squamosa) Disease Image Dataset** Data identification number: 10.17632/jtgh2885yf.2 Direct URL to data: https://data.mendeley.com/datasets/jtgh2885yf/2

## 1 Value of the Data

• *Comprehensive and Diverse:* The dataset comprises 8226 high-resolution images, serving as a valuable resource for studying custard apple fruit and leaf diseases. It enables effective disease detection and classification in custard apple.
• *First Open-Access Dataset:* This dataset is the first openly accessible collection of custard apple fruit samples. It facilitates collaboration among researchers, accelerating advancements in disease detection, monitoring, and management in custard apple cultivation.
• *Disease Management:* With 06 categories including 5 fruit disease and 1 leaf disease. The dataset demonstrates both comprehensiveness and diversity. This kind of diversity is essential for testing and training machine learning algorithms, guaranteeing the accuracy of these algorithms in identifying and categorizing various diseases of custard apple tree.
• *Precision Agriculture Applications: The dataset's applicability with machine learning algorithms enables development of automated disease identification systems in precision agriculture system.* With 8226 images, researchers can develop and evaluate models using deep learning, feature extraction, and pattern recognition, enhancing disease detection accuracy. In the scientific field of plant pathology, it promotes collaborations and novel approaches.

This Custard Apple dataset is a valuable resource with applications in research, agriculture, technology development, and disease detection.

## 2 Background

Custard apple, also known as Sugar apple, is a subtropical fruit in the Annonacea family, recognized by its tough green skin and creamy interior with various shapes. Its sweet, granular flesh contains numerous seeds and is rich in fibers, minerals, and vitamins, offering health benefits. The fiber aids digestion, prevents constipation, and supports detoxification. Packed with antioxidants like flavonoids, phenolic compounds, kaurenoic acid, and vitamin C, custard apple fights free radicals associated with chronic diseases, cancer, and heart conditions. Lutein and carotenoid antioxidants protect eyes from oxidative damage, reducing the risk of age-related macular degeneration, vision loss, and cataracts.

The development of the Custard Apple Leaf and Fruit Disease Image Dataset was inspired by the inherent medicinal properties and economic significance in the food and pulp making industry. Given its medicinal applications and substantial market value, industries demand top-notch custard apples. The health and quality of these plant components are pivotal for fruit-based food industries. However, diseases can impede fruit production. Therefore, the identification of diseases becomes crucial. To address this need, we formulated a disease dataset for both fruit and leaf, encompassing various types of diseases. This initiative not only caters to the interests of food industries seeking superior fruit quality but also benefits farmers by providing a tool for evaluating the quality in their cultivation practices.

## 3 Data Description

The image datasets play a crucial role in various fields, ranging from computer vision and machine learning to medical research and social sciences. These datasets provide a rich source of visual information that enables researchers, developers, and professionals to train and validate their models, algorithms, and theories. By having access to diverse and well-curated image datasets, researchers can explore new possibilities, enhance the accuracy and robustness of their models, and gain valuable insights into patterns, trends, and relationships within the visual data.

An image dataset specific to custard apple fruit and leaf diseases holds significant importance in the agricultural domain. Such datasets provide researchers, agronomists, and farmers with a valuable resource to identify, classify, and study various fruit and leaf diseases.. By analysing these images, experts can develop more accurate disease detection algorithms and early warning systems. This aids in prompt disease management, preventing widespread crop damage and yield loss. Additionally, a comprehensive dataset allows for the exploration of disease patterns, environmental factors, and potential mitigation strategies. In summary, a Custard apple fruit and leaf disease image dataset plays a pivotal role in advancing research, improving crop management practices, and ensuring the overall health and productivity of custard apple.

**Fig. 1:**
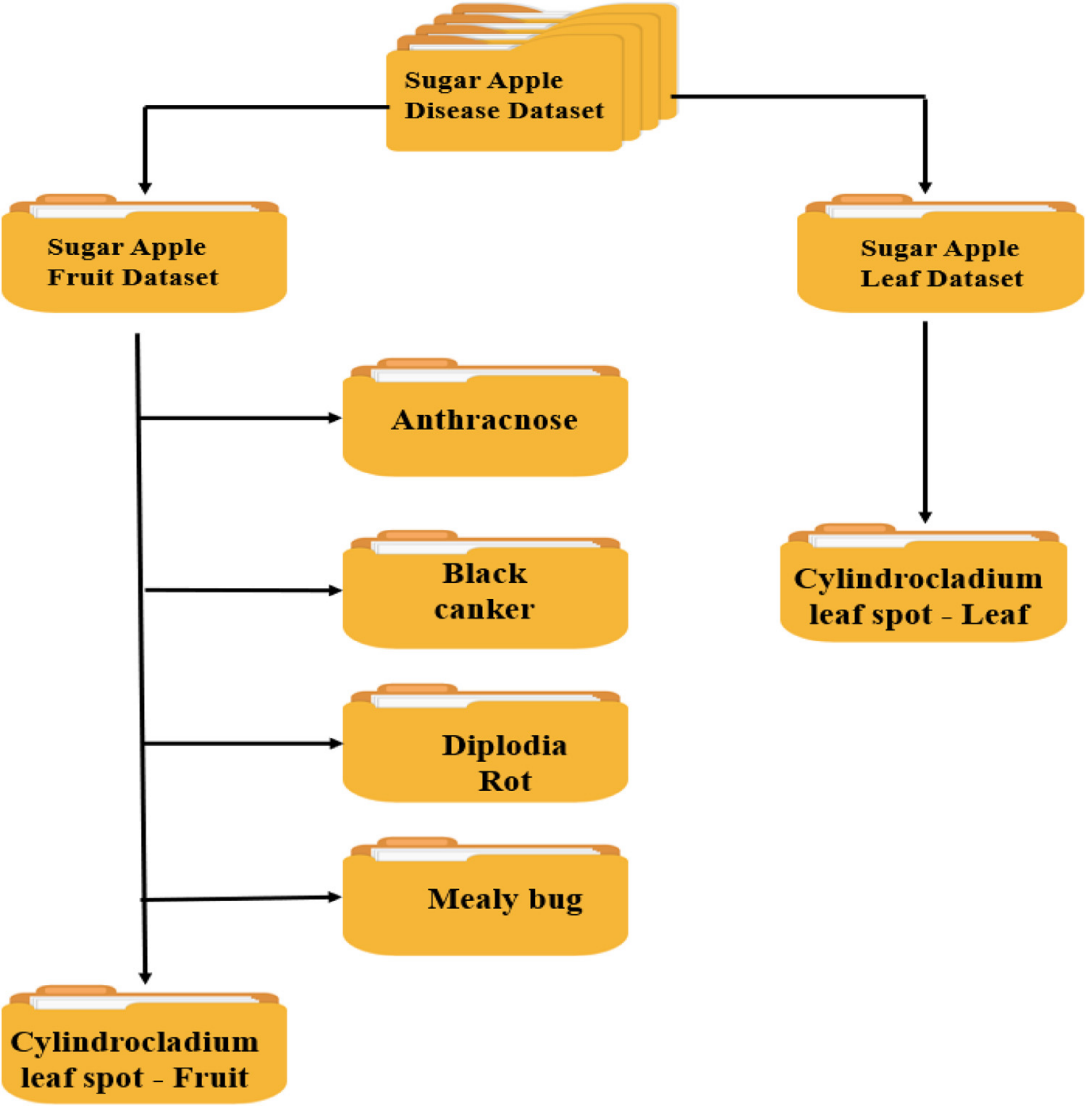
Arrangement of the custard apple disease dataset's folders.

This Custard Apple fruit and leaf Dataset [1] contains a diverse collection of 8226 high-resolution images. The images are stored in JPEG format and have dimensions of 768 × 1024 pixels. The dataset is categorized into 06 distinct classes, including 1 leaf disease category and 5 fruit disease categories. The disease categories cover a range of common custard apple fruit disease, such as Athracnose, Blank Canker, Diplodia Rot, Leaf Spot on fruit, and Mealy Bug (Table 1). It also covers common leaf disease Leaf spot (Table 1). Each category is labelled and organized in separate folders, ensuring easy access and identification of specific disease samples. The Fig. 1 shows directory structure of custard apple disease dataset. The images were collected through extensive field surveys conducted in Custard apple growing regions. The data collection process involved using quality cameras to capture images from various angles, including both sides of the fruit and leaves. Images were taken in the field and by cutting/separating individual fruit and leaves, capturing different stages and manifestations of the diseases. This approach ensures a comprehensive representation of the visual characteristics of custard apple diseases within the dataset. The dataset's images are of high quality, with a resolution set at 72 dots per inch (dpi), ensuring clear and detailed visual representation of the custard apple disease samples.

**Table 1:** Quantitative breakdown: image count per custard apple disease category.

Disease name	Total images
Anthracnose	1075
Black canker	1780
Cylindrocladium leaf spot -fruit	867
Cylindrocladium leaf spot- Leaf	1255
Diplodia rot	1645
Mealy bug	1604
**Total**	**8226**

Table 1 shows Custard Apple disease dataset with its categories and count per category. Fig. 1 illustrates Custard Apple Disease dataset folder structure. Table 2 shows exemplar images from the dataset.

**Table 2:** Sample images of different custard apple diseases.

Prior research has extensively explored disease detection in fruit crops focusing on image-based classification and machine learning algorithms [[2], [3], [4], [5], [6], [7], [8], [9], [10]]. This dataset's main goal is to offer a thorough selection of high-resolution images of custard apple fruit and leaves that cover a range of diseases. The dataset is to aid in the development and assessment of machine learning methods and algorithms for custard apple disease detection and classification. The scientific community is encouraged to collaborate and share information via the open availability of this dataset. The primary aim of this dataset is to facilitate the development of efficient algorithms for disease detection and classification, enhance disease management strategies, and eventually aid in the sustainable production of custard apples by researchers and practitioners.

## 4 Experimental Design, Materials and Methods

### 4.1 Experimental design

**Fig 2:**
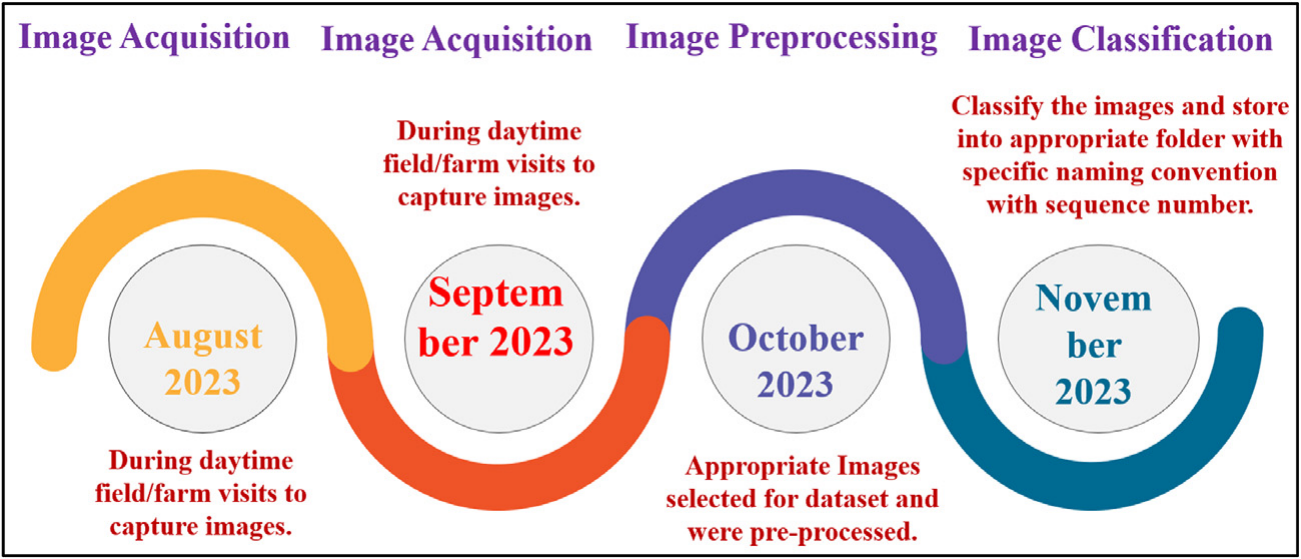
Data acquisition phase.

The Custard Apple Disease dataset was generated through the acquistion of images using high resolution rear cameras of Samsung F23 5 G Mobile. The Fig. 2 provides a summary of the data acquisition steps undertaken for the project.

**Step 1: Image Acqusition Phase (Duration August to September):** In this phase, We performed field visits in daylight to capture photographs depicting different diseases affecting custard apple fruits and leaves, aiming to compile a thorough collection of images related to these diseases.

**Step 2: Image Preprocessing Phase (Duration- October):** During this phase, the collected images underwent a review process, and suitable ones for the dataset were chosen. The selected images were then subjected to pre-processing, which could involve actions such as resizing, cropping, and enhancing as required.

**Fig. 3:**
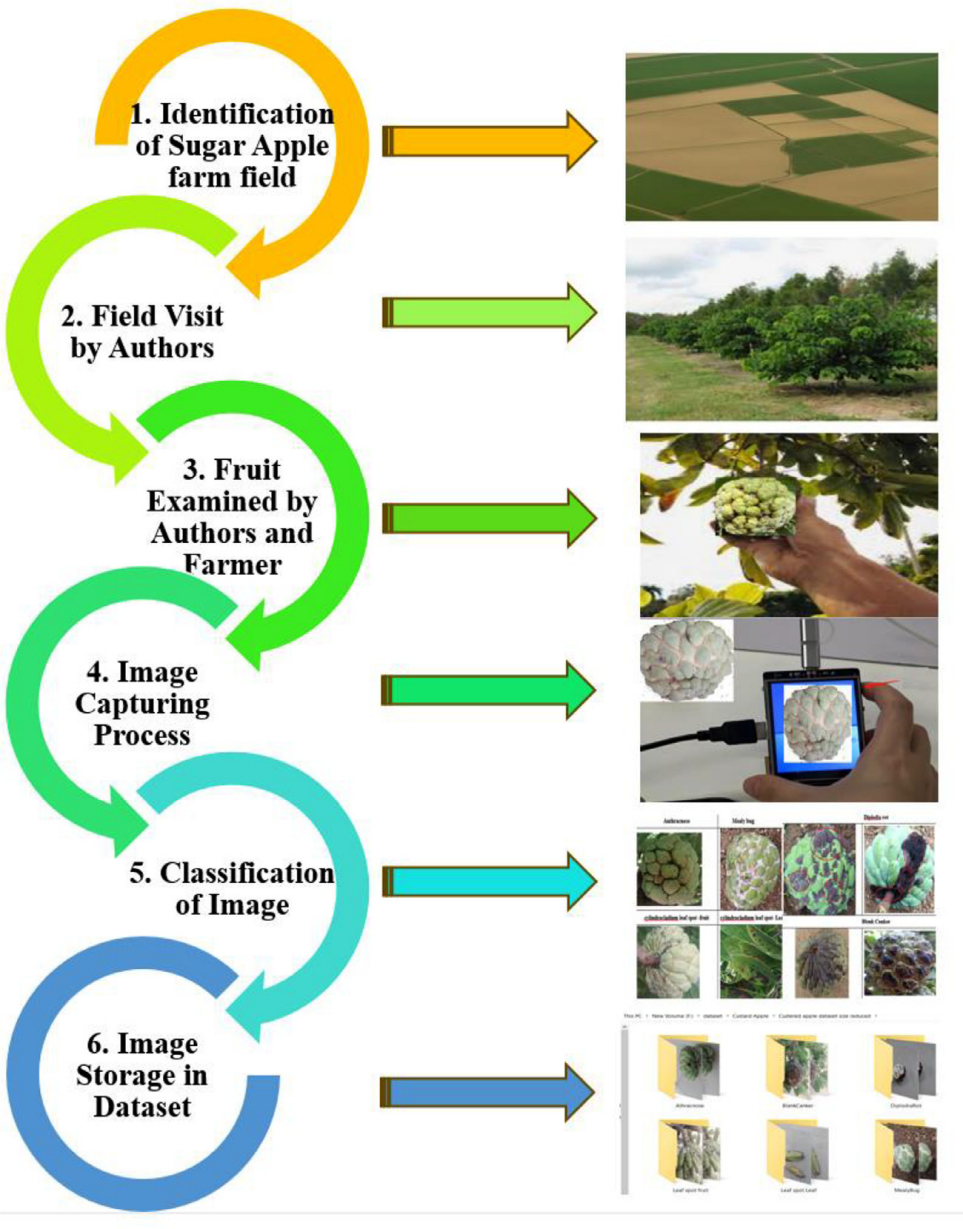
Stage-by-stage progress: dataset collection with visual samples.

**Step 3: Image Classification (Duration- November):** Image classification is essential process to create set of specific disease dataset which includes batch conversion with labelling the image with a specific sequence number. Fig. 3 shows stepwise Classification of images. Table 3 shows image classification parameters for dataset images.

**Table 3:** Key image classification metrics for disease dataset.

Image Classification parameter	Description
Quality of Image	Image is converted into good quality and low quality
EXIF Data	A standard called exchangeable image file format describes the file types that digital cameras or smartphone can use for their images, sounds, and auxiliary information.
XMP data	An XML-based metadata format called XMP data is used to describe a file's contents.
IPFC data	``International Press Telecommunications Council'' is what IPTC stands for. A standardized metadata format known as IPTC data was developed specifically for media and news agencies to use. Title, description, and location are among the image details that are included.

**Step 1:** create a dataset of each disease and allocate all the images within specific dataset

**Step 2:** Use Image processing and classification software IrfanView 64bit version 4.62. It is used for batch conversion of disease specific image. Batch conversion is a very important process for classification of images.

**Step 3:** Configuration of images with specific parameter is important step for batch conversion.

In batch conversion it consider following parameters.

### 4.2 Data collection phases

Fig. 3 illustrates the sequential progression of steps involved in curating the dataset, from field identification to dataset compilation. It delineates the six key stages involved in the dataset collection process:

1. Identification of Sugar Apple Farm Field: The initial stage involves pinpointing the sugar apple farm fields relevant to the dataset collection.
2. Field Visits by Authors: Authors conduct on-site visits to the identified fields for data collection purposes.
3. Fruit Examination by Authors and Farmers: Authors collaborate with farmers to examine the sugar apple fruit, inspecting for disease manifestations and other relevant characteristics.
4. Image Capture Process: High-quality images of the sugar apple fruit are captured using appropriate imaging techniques during field visits.
5. Classification of Images: The captured images undergo a classification process to categorize them based on disease presence or other relevant attributes.
6. Image Storage in Dataset: The classified images are then stored systematically within the dataset for further analysis and research purposes.

### 4.3 Materials or specification of image acquisition system

The cameras used in the data acquisition process and the specifications of the captured im- ages:

Samsung Galaxy F 23 5 G Android Mobile:

• Make and Model: Samsung Galaxy F 23 5 G (SM-E236B) Android Mobile.
• Rear Primary Camera: Equipped with a 50-megapixel (f/1.8) lens.
• Camera Sensor: Utilizes the Sony IMX 582 1/2″ sensor.
• Battery: Comes with a 50 0 0 mAh battery.

During the data collection process, efforts were made to adhere to standardized image acquisition practices, capturing each image using the rear cameras of a Samsung F23 5 G Mo- bile known for its high-resolution imaging capabilities. This maintained consistency and quality throughout the dataset. The captured images were saved in JPG format and resized to a resolution of 768 × 1024 pixels. To reduce errors and improve dependability, this multi-step labeling procedure involves careful examination and cross-validation by several experts in the field. In addition to giving the dataset more legitimacy, the validation of disease categories by subject matter experts guaranteed that each disease's visual attributes were accurately represented.

### 3.4 Methods

The dataset was collected through field visits to sugar apple farms. Authors collaborated with farmers to examine and capture high-resolution images of sugar apple fruit. These images underwent detailed classification based on disease presence and other attributes, forming a systematically organized dataset for analysis and research. In order to gather information for the custard apple tree disease dataset, a farm in *Nimgaon Bhogi, Taluka-Shirur, District-Pune, India* was visited. Images were taken in a variety of settings, such as with fruits and leaves in their natural state and after they were cut off or separated from the plant. This made it possible to depict custard apple diseases in all of their variations in a thorough manner.

## Limitations

The dataset is collected from a specific region, potentially limiting its applicability to other geographical areas with different disease prevalence or manifestations.

## Ethics Statement

Our study does not involve studies with animals or humans. Therefore, we confirm that our research strictly adheres to the guidelines for authors provided by Data in Brief terms of ethical considerations.

## CRediT authorship contribution statement

**Sandip Thite:** Conceptualization, Supervision, Writing – review & editing. **Kailas Patil:** Methodology, Data curation, Writing – original draft. **Rohini Jadhav:** Writing – original draft, Writing – review & editing. **Yogesh Suryawanshi:** Conceptualization, Writing – review & editing. **Prawit Chumchu:** Writing – review & editing.

## Data Availability

Sugar Apples / Custard Apples (Annona squamosa) Disease Image Dataset (Original data) (Mendeley Data)
